# An emerging trend of rapid increase of leukemia but not all cancers in the aging population in the United States

**DOI:** 10.1038/s41598-019-48445-1

**Published:** 2019-08-19

**Authors:** Taisen Hao, Min Li-Talley, Alison Buck, WenYong Chen

**Affiliations:** 1 Department of Cancer Biology, Beckman Research Institute, City of Hope, Duarte, CA 91010 USA; 2 Department of Biostatistics, Beckman Research Institute, City of Hope, Duarte, CA 91010 USA; 3 Department of Biology, Biola University, La Mirada, CA 90638 USA

**Keywords:** Cancer epidemiology, Cancer epidemiology

## Abstract

The “baby boomers” born in 1946–1964 in the United States (U.S.) started to reach the age of 65 in 2011, rapidly accelerating U.S. population aging. There are great public concerns about its impact on health care with anticipation of rising cancer incidences. We examined the incidences and deaths of leukemia and overall cancer in the U.S. from 1998 to 2018. The acute myeloid leukemia (AML) and chronic myeloid leukemia (CML) incidences remained constant prior to 2011 but have climbed up substantially since then, and the chronic lymphocytic leukemia (CLL) incidence has increased continuously since 1998. The significant increase of myeloid leukemia and CLL incidences was strongly correlated with the U.S. population aging. The incidence of all cancers was increased in correlation with a small increase in aging population prior to 2011, but surprisingly has changed marginally since 2011, which was not significantly correlated with the accelerated population aging. We observed the most substantial decline of deaths with CML, whereas AML deaths continued to rise in the past 20 years. In conclusion, the overall cancer incidence was not increased as fast as previously feared with aging Americans; however, the incidences of myeloid leukemia and CLL significantly outpaced that of all cancers.

## Introduction

Leukemia is a collection of several malignancies derived from the pathological alterations of leukocytes in the blood system and blood-forming organs. Overall, leukemia is estimated to account for about 3.5% of all cancer incidences and 4% of cancer-derived mortalities in the United States^1^. Based on the origin of the predominant cell type (myeloid or lymphoid) and the rate of disease progression (acute or chronic), leukemia is categorized into four major subtypes: acute myelogenous leukemia (AML), chronic myelogenous leukemia (CML), acute lymphocytic leukemia (ALL), and chronic lymphocytic leukemia (CLL). Worldwide, the leukemia incidence has stayed relatively stable over the years, but regional variations have been spotted in different geological areas owing to the differences in ethnicity, environmental factors, and life styles^2–8^. Progress has been made in recent years for the treatment of certain types of leukemia. CML, for instance, is a disease caused by a reciprocal translocation between the long arms of chromosomes 9 and 22, forming a Philadelphia chromosome that produces a hybrid oncogenic BCR-ABL1 tyrosine kinase to drive uncontrolled cellular proliferation^9^. The tyrosine kinase inhibitor imatinib methylate was approved by the U.S. FDA as the first line CML treatment in 2001 and has since effectively controlled the disease, raising the five-year survival rate of CML to close to 90% from 30% in the pre-imatinib era^10^.

Age is a major risk factor for human cancer^11,12^. Previous epidemiological studies based on age-period-cohort models concluded that cancer incidence including leukemia increases dramatically with age and peaks at 80 to 85^12–16^. Except for ALL that is the most common childhood cancer, AML, CML, and CLL are all age-dependent with the median age at diagnosis around 65 to 72^17–19^. However, cancer incidence drops in the oldest old population above age 85^20^. Historically, aging population was relatively stable due to a constant birth rate, and very few epidemiological studies were able to provide convincing data to demonstrate the relationship between large population aging and cancer burden due to insufficient data source^21,22^.

The baby boomer generation generally refers to the post-World War II birth from mid-1946 to mid-1964, during which the U.S. population experienced exponential growth by virtue of the continuing increase of new births^23^. By mid-2011, the baby boomer generation started to reach 65, resulting in an age-related demographic change in the U.S. population^23^. By 2030, all “baby boomers” will turn 65 and above, and the oldest baby boomers will reach 85, which is the peak epidemiological age for cancer incidence^12–16^. The accelerated population aging by “baby boomers” raised national concerns about its impact on the American health care in 1990s to 2000s, especially with anticipation of a high rise of cancer incidences^24,25^. It is therefore important to know the actual impact of the accelerated population aging on leukemia and overall cancer incidences in the U.S. Although the aging population will continue to expand until 2030, we are now nearly halfway into this major demographic change of the population, which would allow us to assess its early impact on cancer. Here we examined the incidences and deaths of leukemia and all cancers in the U.S. in the past 20 years, and determined how the increased U.S. population aging affected leukemia and all cancers.

## Results

### The number of cases and deaths of leukemia and all cancers

**Figure 1:**
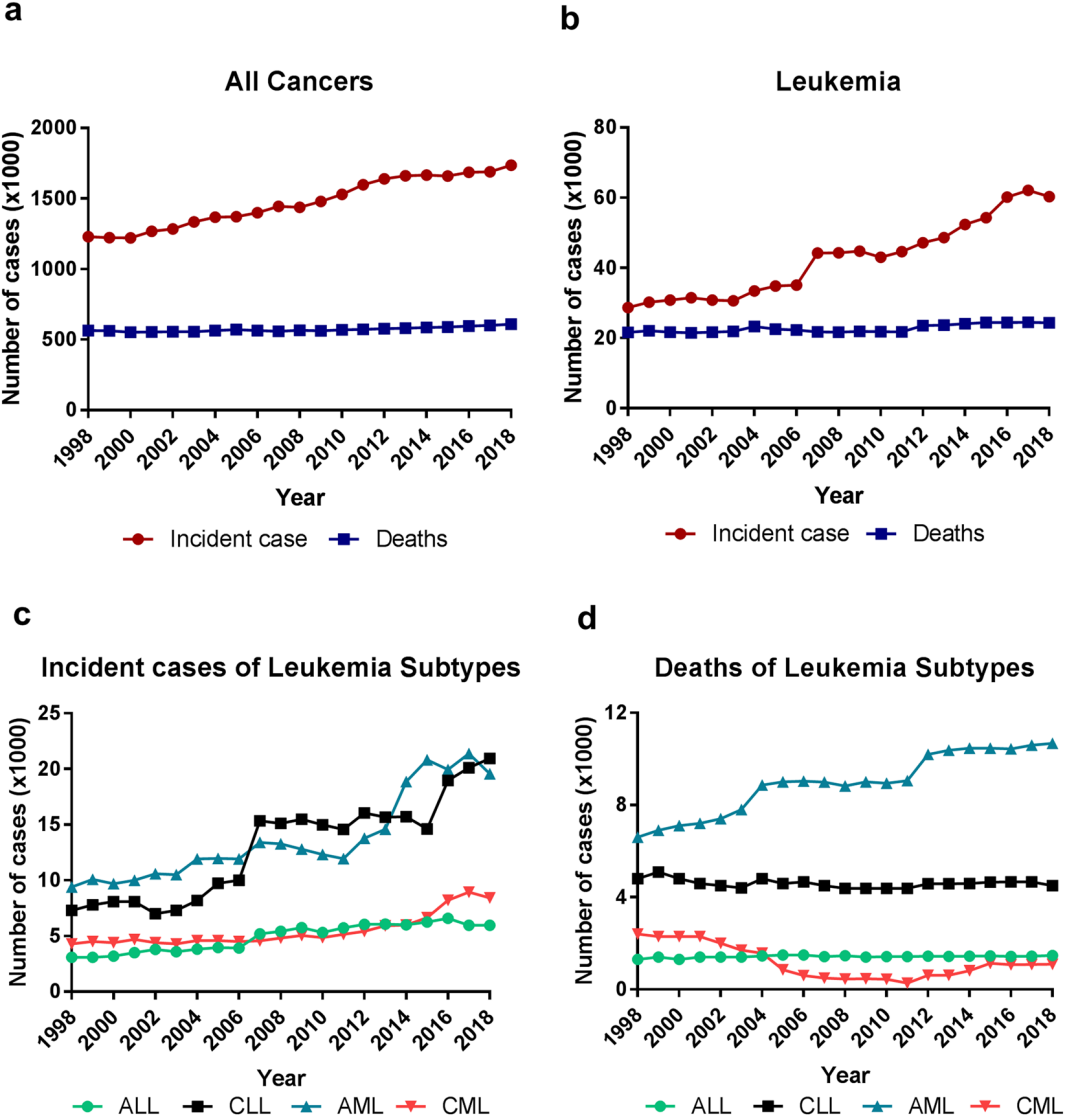
The incident cases and deaths of leukemia and all cancers in the United States from 1998 to 2018. (**a**,**b**) Incident cases and deaths of all cancers (**a**) and leukemia (**b**). (**c**) Incident cases of four major subtypes of leukemia. (**d**) Deaths of four subtypes of leukemia.

Using the cancer statistics data collected by the American Cancer Society (ACS)^1,26–45^, we analyzed the changes in the number of cases and deaths of leukemia and all cancers in the U.S. from 1998 to 2018. The number of cases of all cancers has been increasing steadily over the years, from 1,228,600 cases in 1998 to 1,735,350 in 2018, up 41%. The cancer deaths slightly increased from 564,800 cases in 1998 to 609,640 in 2018 (Fig. 1a). Compared to all cancers, leukemia cases have been increasing much faster, from 28,700 cases in 1998 to 60,300 in 2018, up 110%, with an abrupt increase between the year 2006 and 2007. Like all cancer deaths, leukemia deaths slightly increased from 21,600 cases in 1998 to 24,370 in 2018 (Fig. 1b).

The four major subtypes of leukemia, CML, AML, CLL and ALL, showed distinct distribution of incident cases and deaths, with CLL and AML increasing most rapidly in both the numbers of cases and percentages. The cases of CLL increased dramatically from 7,300 cases in 1998 to 20,940 in 2018, up 187% (Fig. 1c). This was followed by AML that increased from 9,400 cases in 1998 to 19,580 in 2018, up 108%. CML cases increased from 4,300 cases in 1998 to 8430 in 2018, up 96%; whereas ALL increased from 3100 in 1998 to 5,960 in 2018, up 92% (Fig. 1c). The AML deaths were the highest and continued to rise over the years from 6,600 cases in 1998 to 10,670 in 2018, up 62% (Fig. 1d). The deaths of CLL and ALL remained relatively constant in the past 20 years. Notably, CML displayed a substantial reduction in deaths from 2,400 cases in 1998 to the lowest point 270 in 2011 (down 90%), but CML deaths resumed to increase to 1,090 cases in 2018 (Fig. 1d).

### Population-adjusted leukemia and other cancer incidences and deaths

**Figure 2:**
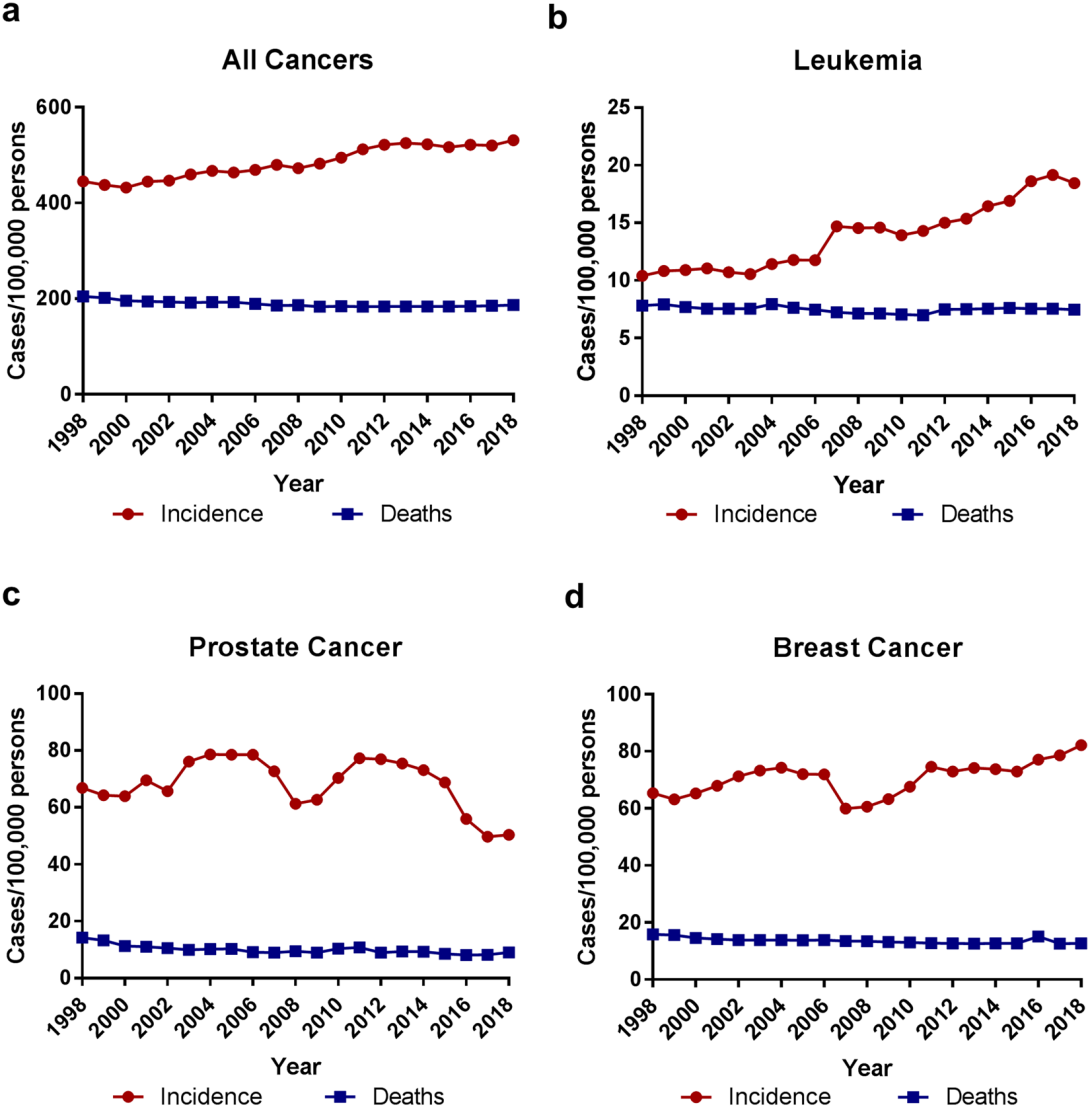
The population-adjusted incidences and deaths of leukemia and other cancers in the United States from 1998 to 2018. Comparison of population-adjusted incidences and deaths of all cancers (**a**) vs leukemia (**b**), prostate cancer (**c**), and breast cancer (**d**). The rates for incidences and deaths were normalized to total population in each year.

To more accurately analyze the changes of leukemia and all cancer incidences and deaths, we normalized the above data to the total population of the United States of each corresponding year. All incidences and death rates presented hereafter are described as the number of cases per 100,000 persons unless specified. The all cancer incidence increased moderately over the years from 445.3 cases in 1998 to 531.1 cases per 100,000 persons in 2018, up 19%. The rate of all cancer deaths exhibited a slight reduction from 204.7 cases in 1998 to 186.6 in 2018, down 9% (Fig. 2a). In contrast, the leukemia incidence increased much faster from 10.4 in 1998 to 18.5 in 2018, up 78%. Like all cancer deaths, the rate of leukemia deaths showed a slight reduction from 7.8 in 1998 to 7.5 per 100,000 persons in 2018, down 4% (Fig. 2b).

We further compared two common solid tumors, prostate and breast cancers, with leukemia. The prostate cancer incidence fluctuated in the past 20 years with two major drops in 2008 and 2017 (Fig. 2c). Overall, the prostate cancer rate decreased significantly from 66.9 in 1998 to 50.4 per 100,000 persons in 2018, down 25%. The total case numbers of prostate cancer reached the lowest point of 161,360 in 2017, about 50% reduction from that in 1998^45^. The prostate cancer death rate has also been decreasing steadily over time, from 14.2 in 1998 to 9.0 per 100,000 persons in 2018, down 37% (Fig. 2c). The breast cancer incidence elevated from 65.3 in 1998 to 82.2 in 2018, up 26%, with some fluctuations especially a notable drop in the year 2007 (Fig. 2d). However, the breast cancer death rate has decelerated from 15.9 in 1998 to 12.7 in 2018, down 20% (Fig. 2d). Therefore, the population-adjusted rates of prostate and breast cancer deaths were reduced much more than that of leukemia. In contrast to the rapid increase of the leukemia incidence, the population-adjusted prostate cancer incidence was reduced remarkably and the breast cancer incidence was increased at a much slower pace.

**Figure 3:**
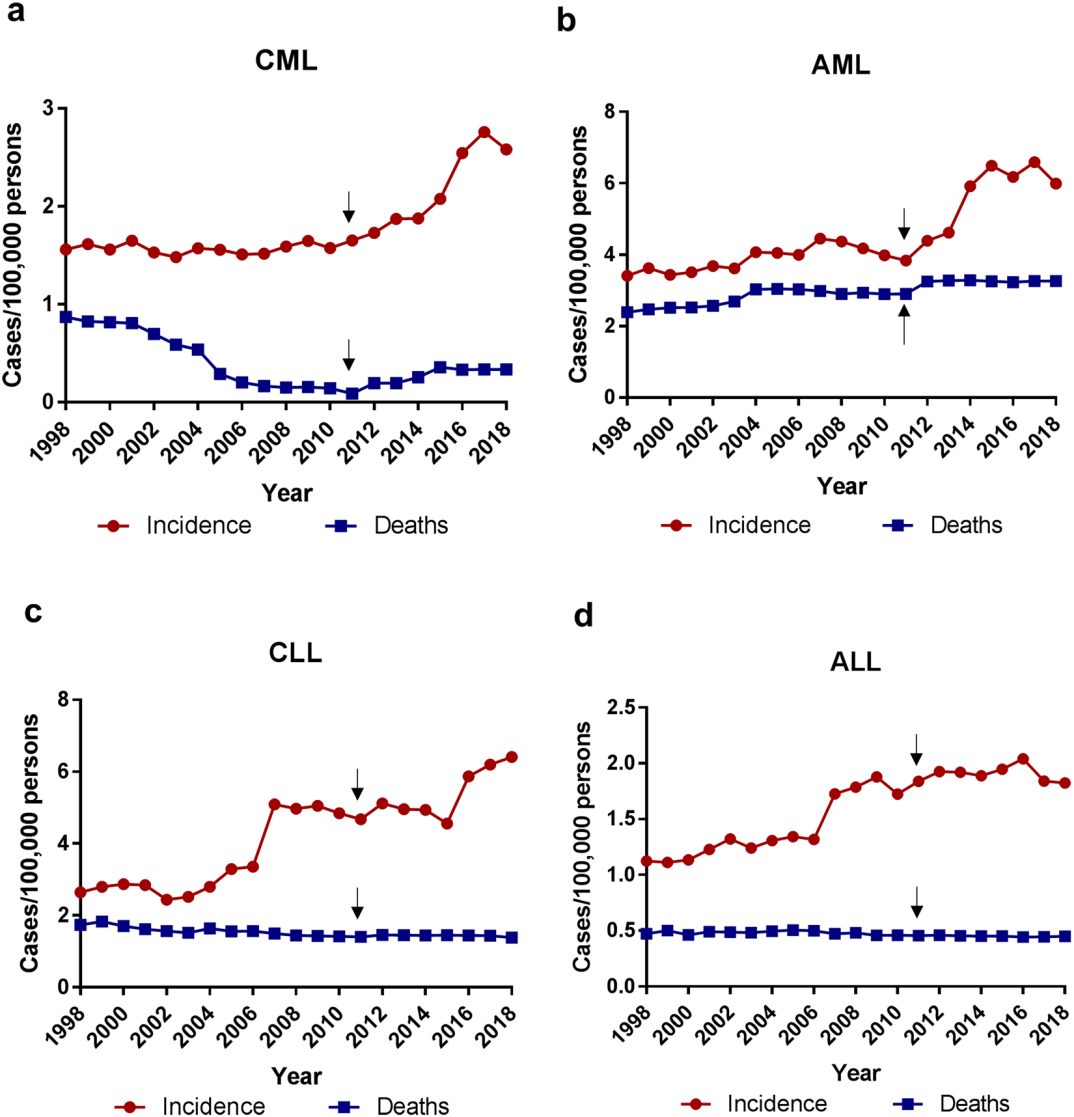
The population-adjusted incidences and deaths of four major subtypes of leukemia. The arrows indicate year 2011 that displayed the lowest CML death rate and marked the turn for rapid increase of myeloid leukemia.

We then examined the four subtypes of leukemia. The CML incidence remained relatively constant from 1998 to 2011 at ~1.6 cases per 100,000 persons as previously known^9^, but increased rapidly since then to 2.6 in 2018, up 62% (Fig. 3a). The AML incidence fluctuated moderately from 1998 to 2011, followed by the largest escalation over 31% from 2012 to 2018. The overall rate of AML increased from 3.4 cases per 100,000 persons in 1998 to 6.0 in 2018, up 76%, mostly after 2011 (Fig. 3b). These data suggested an important trend of rapid increase of myeloid leukemia after 2011. Similar to those described in Fig. 1d, the rate of CML deaths decreased over the years from 0.87 per 100,000 persons in 1998 to 0.33 in 2018, down 62%, with its nadir at 0.087 in 2011 (Fig. 3a). However, the rate of AML deaths rose steadily from 2.4 per 100,000 persons in 1998 to 3.3 in 2018, up 38% (Fig. 3b). For lymphoid leukemia, the CLL incidence increased from 2.6 per 100,000 persons in 1998 to 6.4 in 2018, up 146% (Fig. 3c). There was an abrupt 52% increase in the incidence from 2006 to 2007, and another 29% increase from 2015 to 2016 (Fig. 3c). The rate of CLL deaths decreased overtime from 1.7 per 100,000 persons in 1998 to 1.4 in 2018, down 18%. The ALL incidence rose moderately from 1998 to 2006, and after an abrupt 30% increase from 2006 to 2007, it became relatively flat through 2018 (Fig. 3d). Overall, the ALL incidence increased from 1.1 per 100,000 persons in 1998 to 1.8 in 2018, up 64%. The rate of ALL deaths remained at 0.46 per 100,000 persons for the past 20 years (Fig. 3d). Together, these data suggest that there is a marked increase of leukemia incidence from 1998 to 2018, particularly myeloid leukemia and CLL after 2011, outpacing the increase of all cancers. Depending on the subtypes, leukemia death rates may be less, on par with, or more than other cancers.

### Impact of aging on leukemia and all cancer incidences

**Figure 4:**
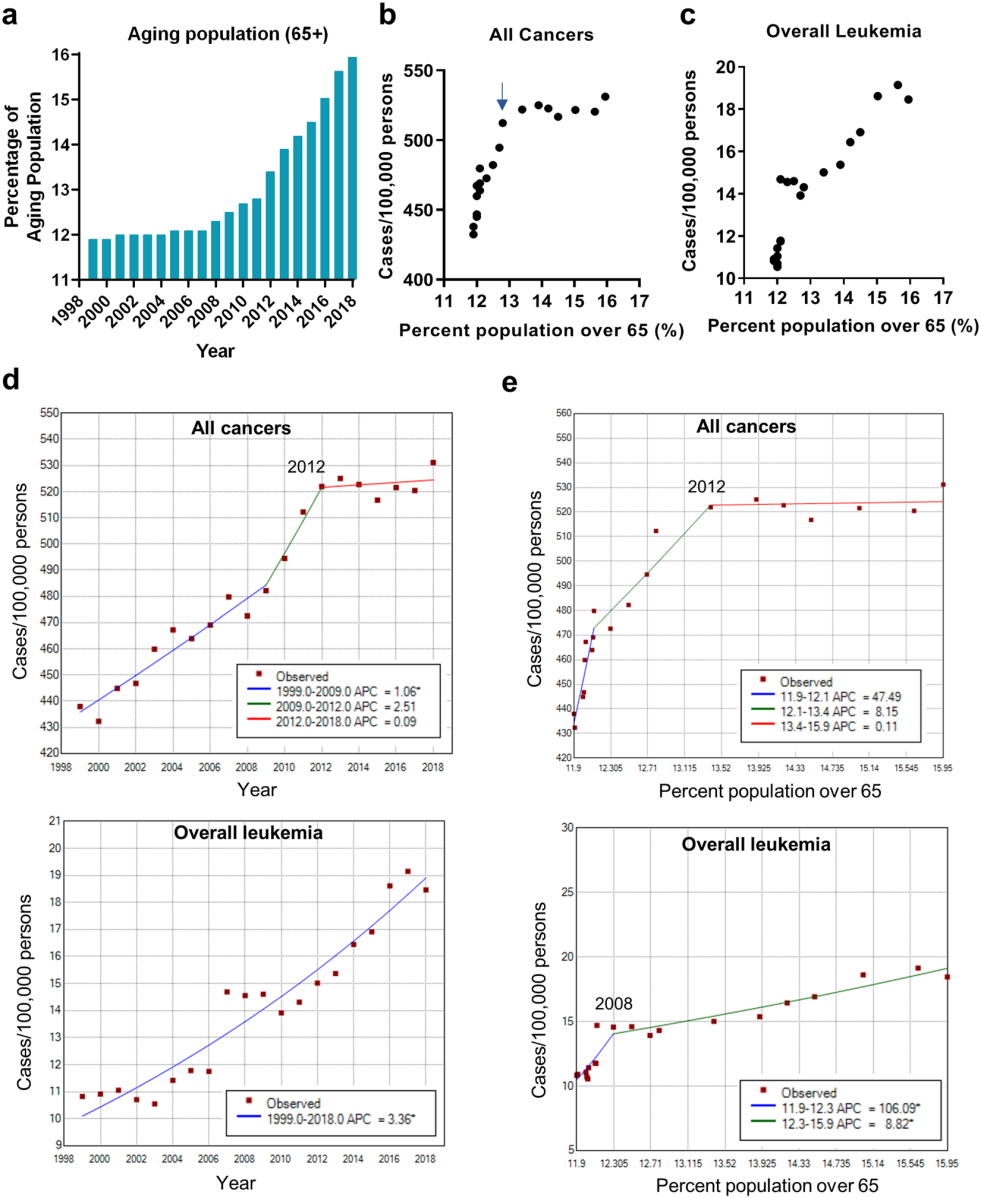
Correlation of the US population aging and cancer incidences. (**a**) The changes of aging population (>65 years) in the United States from 1999 to 2018. (**b**,**c**) Pearson’s correlation analysis of aging population vs all cancers (**b**) and overall leukemia (**c**). The arrow indicates year 2011 that “baby boomers” started to reach the age of 65. (**d**,**e**) Joinpoint regression trend analysis of all cancers and overall leukemia with year (**d**) or percent population over 65 (**e**) as the variable. The years for the last joinpoints were indicated.

**Figure 5:**
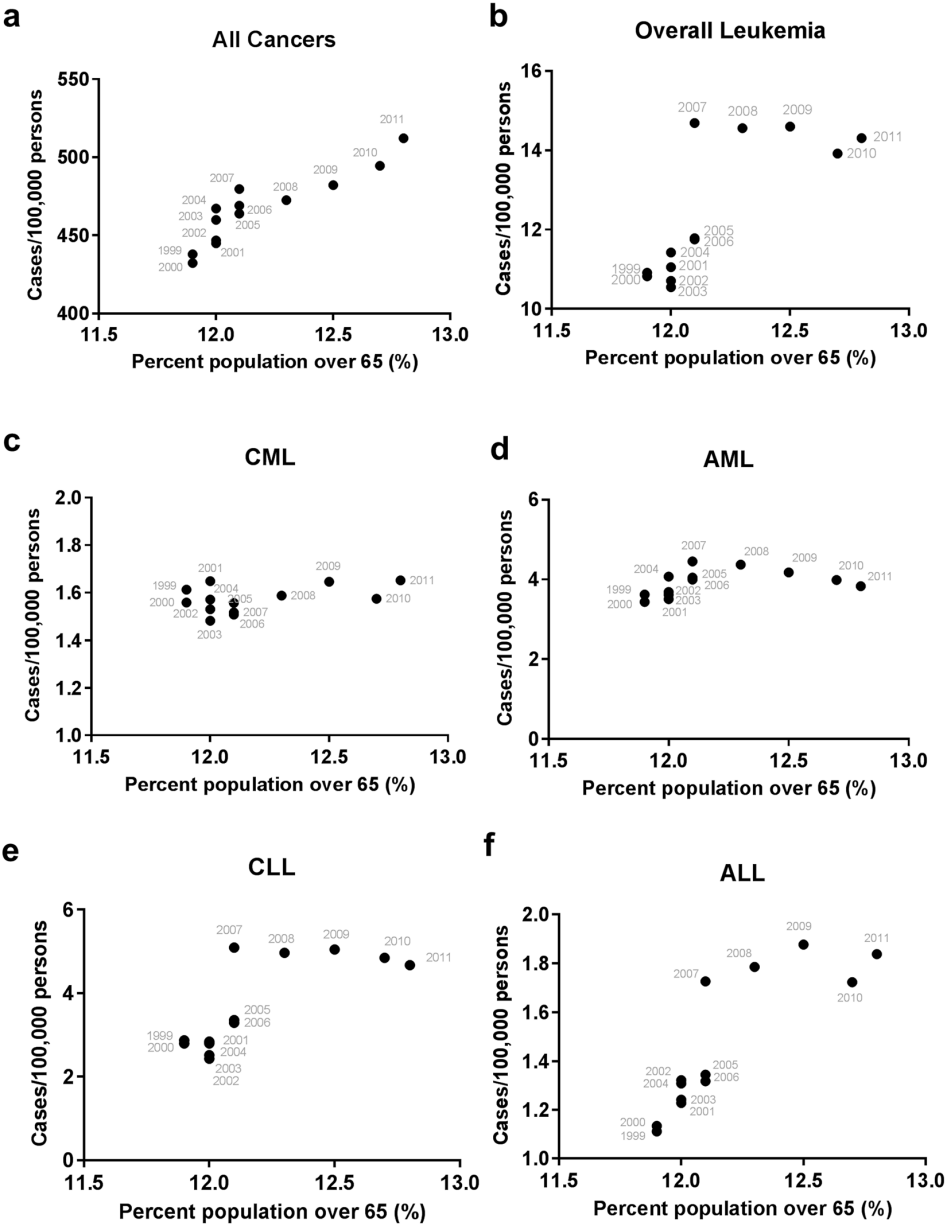
Correlation of the US population aging and cancer incidences before 2011. Pearson’s *r* correlation plots for aging population against (**a**) all cancers, (**b**) overall leukemia, (**c**) CML, (**d**) AML, (**e**) CLL, and (**f**) ALL incidences prior to the year 2011.

**Figure 6:**
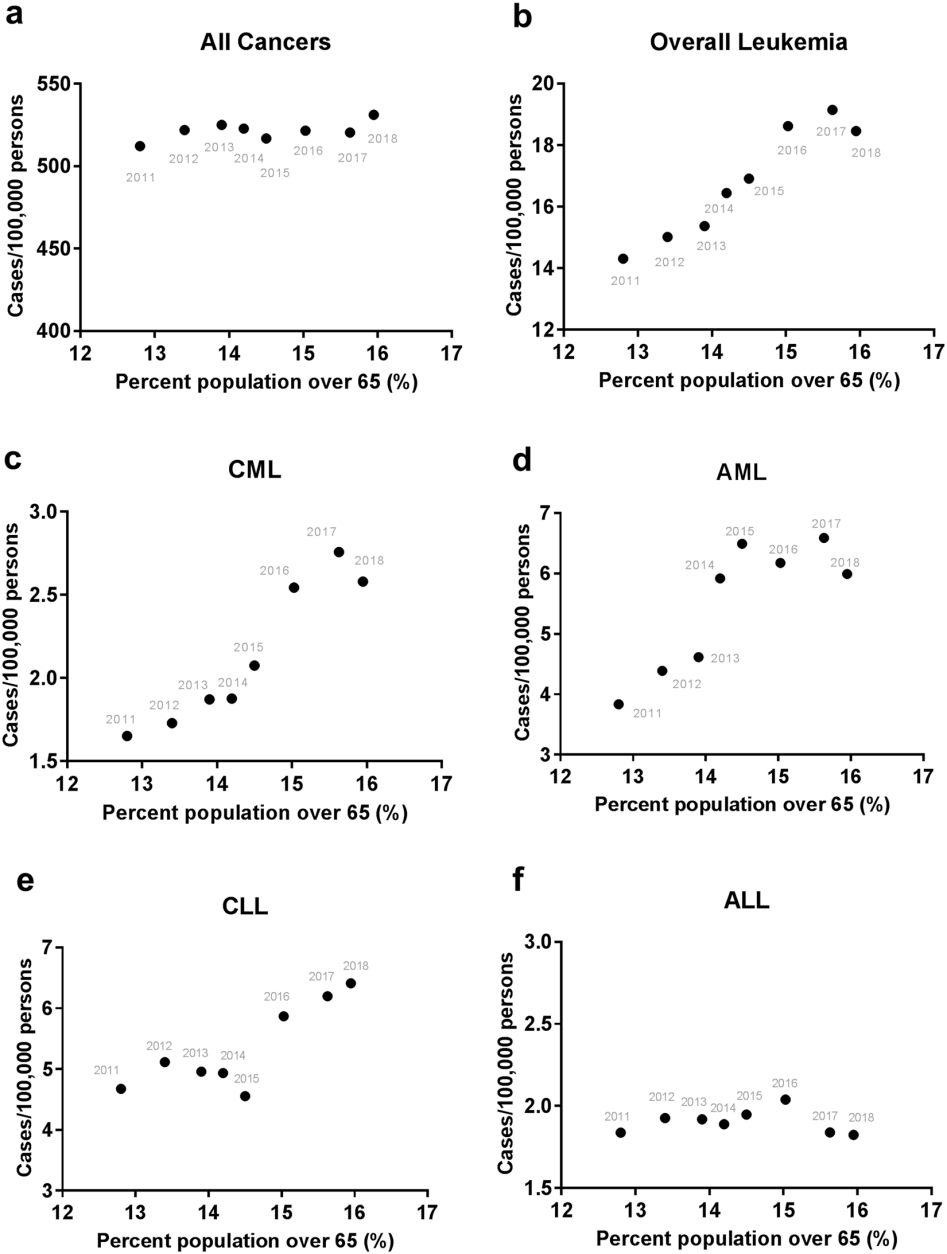
Correlation of the US population aging and cancer incidences after 2011. Pearson’s *r* correlation plots for aging population against (**a**) all cancers, (**b**) overall leukemia, (**c**) CML, (**d**) AML, (**e**) CLL, and (**f**) ALL incidences after the year 2011.

We sought to identify a potential contributing factor to the high rate of leukemia. As the U.S. population undergoes accelerated aging with “baby boomers”, we hypothesized that the increasing aging population in the U.S. may largely contribute to the elevated leukemia incidence for the past several years. As shown in Fig. 4a, the percentage of the population aged over 65 displayed a sharp increase from 12.8% in 2011 to 15.9% in 2018. To test our hypothesis, we performed Pearson’s correlation analysis between the aging population in the U.S. aged over 65 and the corresponding rates of all cancers vs leukemia from 1999 to 2018. The age 65 was used to define the aging population because it is the traditional calendar age for entry to various social, economic, and medical entitlement programs in the U.S., and it also marks the major increase of myeloid leukemia incidences^46^. The aging population data for this period were obtained exclusively from the U.S. Census Bureau. We found that the incidences of all cancers (Fig. 4b) and leukemia (Fig. 4c) showed strong positive correlations with the population aging, and the Pearson’s correlation r values were all highly significant (Table 1). We then split the data into two time periods: 1998 to 2011 and 2011 to 2018. This separation was based on that “baby boomers” started to turn 65 in the mid-2011^23^. The year 2011 was included in both time periods because we could not further segregate the data within the year. We found a rapid increase in the incidence of all cancers from 1999 to 2011 that was strongly correlated with a small increase in the aging population (Fig. 5a and Table 1), but a nearly flat rate of all cancers from 2011 to 2018 that was not significantly correlated with a large increase in the aging population (Fig. 6a and Table 1). In contrast, the incidence of leukemia showed a continuous increase from 1999 to 2018 with some fluctuations, which was strongly correlated with the population aging in both sub-periods, especially from 2011 to 2018 (Figs 4c, 5b, 6b and Table 1). When examining the effect of sex, all cancers incidence continued to rise significantly in females from 2011 to 2018, but decreased in males in association with a significant drop in prostate cancer (Supplementary Table S1).

**Table 1:** Correlation of the percentage of U.S. population over 65 and cancer incidences.

Cancer type	Year 1999–2018		Year 1999–2011		Year 2011–2018	
	Pearson *r*	*P* value	Pearson *r*	*P* value	Pearson *r*	*P* value
All Cancers	0.85	<0.0001	0.90	<0.0001	**0**.**61**	**0**.**11**
Overall Leukemia	0.92	<0.0001	0.77	0.0019	0.96	0.0002
CML	0.95	<0.0001	**0**.**45**	**0**.**12**	0.94	0.0004
AML	0.92	<0.0001	**0**.**36**	**0**.**22**	0.84	0.0082
CLL	0.80	<0.0001	0.77	0.0022	0.84	0.0094
ALL	0.72	0.0004	0.85	0.0003	**−0**.**066**	**0**.**88**

For myeloid leukemia subtypes, the CML and AML incidences prior to the year 2011 that showed minimal changes were not significantly correlated with the population aging (Fig. 5c,d and Table 1). In contrast, the CML and AML rates showed a strong positive correlation with the population aging from 2011 to 2018 (Fig. 6c,d and Table 1). For lymphoid leukemia subtypes, the substantial increase in the CLL and ALL incidences prior to the year 2011 was significantly correlated with the population aging (Fig. 5e,f and Table 1). After the year 2011, the CLL incidence continued to increase in a positive correlation with the population aging, but ALL displayed a flat rate that was not significantly correlated with the population aging (Fig. 6e,f and Table 1). Notably, CLL was the only leukemia subtype that maintained a relatively constant Pearson’s r value and significant correlation with aging in both 1999–2011 and 2011 to 2018 periods (Table 1). Together, these data revealed a surprising but insignificant correlation of the incidence of all cancers vs the population aging in recent years despite the surge of the U.S. aging population; however, the rapid increase in myeloid leukemia and CLL presented strong positive correlations with the population aging. Among leukemia subtypes, the positive correlation of AML with the population aging appeared to be stronger in females relative to males post 2011 (Supplementary Table S1).

**Figure 7:**
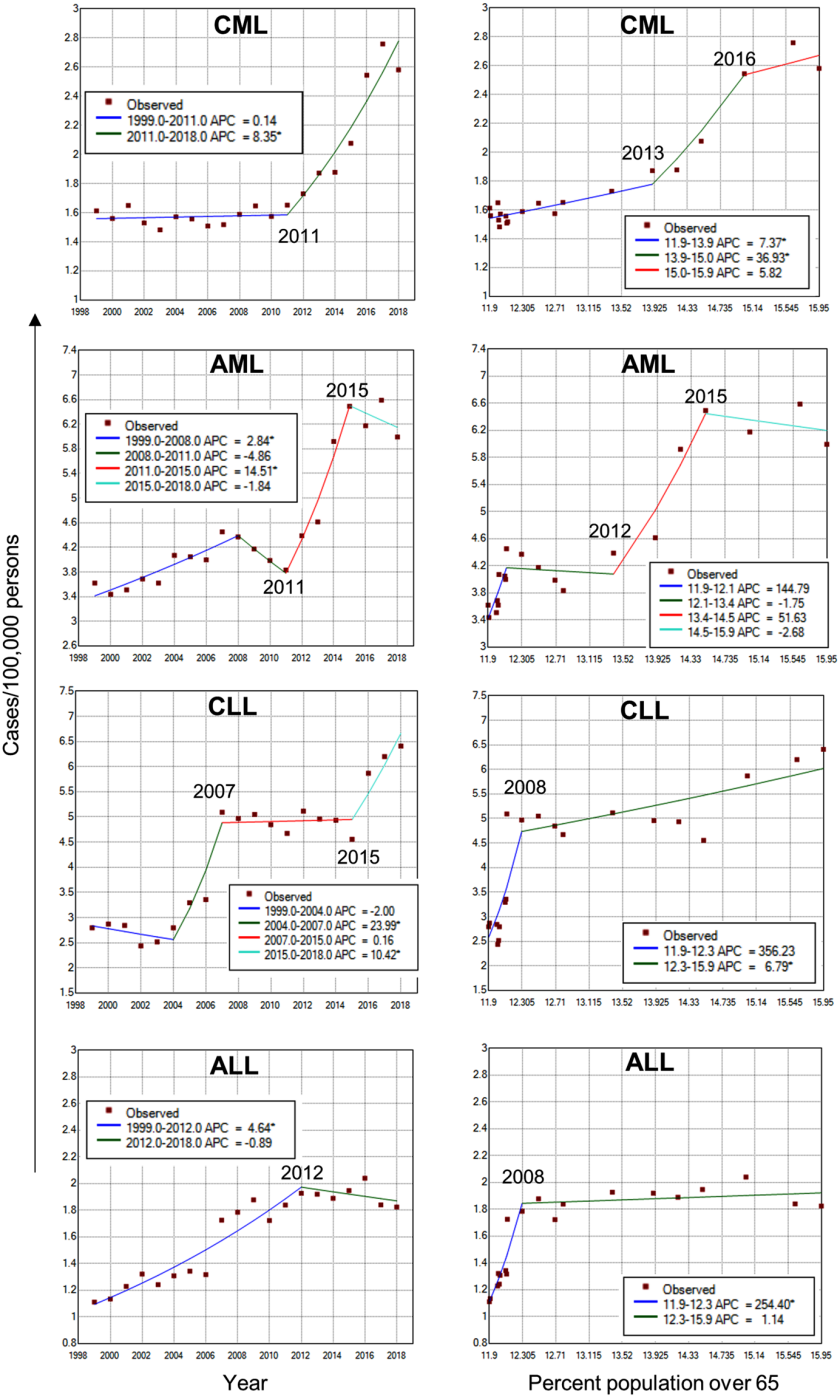
Joinpoint regression analysis of leukemia subtypes. Joinpoint trend analysis of four major leukemia subtypes using the year (left) or the percent population over 65 (right) as the variable. The years for the last two joinpoints were indicated.

To further support the above findings, we performed joinpoint regression trend analyses^47^. Two parallel analyses were carried out using either the percent population over 65 or the year as an independent variable. For all cancers, the annual percent change (APC) was estimated as 0.09 since 2012 when the year was used as an independent variable (Fig. 4d). We observed similar trend when using the percent population over 65 as an independent variable (Fig. 4e), showing that when the percent population over 65 was between 13.4–15.9 (year 2012–2018), the incidence of all cancers increased by 0.11% with every 1% increase in older population over 65. The APC for all cancers incidence during 2012–2018 was not significantly different from 0, which meant that all cancers incidence did not show a significant increase for the aging population between 2012–2018. However, the incidence of overall leukemia showed a completely different trend. The joinpoint analysis identified no joinpoints from 1998 to 2018 and the incidence of leukemia continued rising during this period with APC = 3.36 (p < 0.0001, Fig. 4d). When using the percent population over 65 as an independent variable, one joinpoint was identified. When the older population was between 12.3–15.9% (year 2008–2018), each 1% increase in the older population corresponded to an 8.82% (p < 0.0001) increase in overall leukemia incidence (Fig. 4e). The joinpoint trend analysis for four leukemia subtypes showed different trend patterns, and the year 2011 was identified as a joinpoint for substantially increased CML and AML (Fig. 7). These results were consistent with Pearson’s correlation analysis described above.

Although the ACS data covered the entire U.S. population, they were projective numbers calculated with the National Cancer Institute’s (NCI’s) epidemiology simulation models, which closely matched the final registered cases with about 10% variations^1,48^. The actual registered cases were 2 to 4 years lagging behind those projections^1,48^. We thus examined if the above trends could be observed in the actual cancer data collected by the NCI SEER (Surveillance, Epidemiology, and End Results) program that covered a much smaller U.S. population^46^. We found an increase in overall leukemia incidence, but a decrease of all cancers in the SEER9 data (Supplementary Fig. S1a, b), and the scales of individual leukemia incidence changes were much smaller (Supplementary Fig. S1c,d). The joinpoint trend changes of leukemia in the SEER program were highly dependent on the population size as evidenced by SEER9, 13 and 21 data released in 2018 and 2019 (Supplementary Table S2). In the latest SEER21 with the largest population covered in the program, APC of all cancers was decreased with a substantial reduction of prostate cancer; however, APC of overall leukemia did not change, and APC of CML and AML increased regardless of sex or race from 2007 to 2016 (Supplementary Table S3). Therefore, although the results from SEER data were not quite the same as those from ACS data likely due to the large difference in population size, leukemia, especially myeloid leukemia, relative to all cancers displayed similar moving trends.

## Discussion

Over the past 20 years, the number of leukemia cases in the United States has been steadily climbing up to double the number of 20 years ago. The incidences of AML and CML have remained almost constant prior to 2011. However, a rapid increase in the incidences of these leukemia types was achieved within a relatively short period since 2011, and it was positively correlated with the accelerated U.S. population aging due to “baby boomers”. For lymphoid leukemia, the CLL incidence also exhibited a similar correlation with the U.S. population aging. However, all cancers and ALL incidences showed no significant change or correlation with the U.S. population aging since 2011, which was a surprising finding. Similar to all cancers and ALL, the incidences of breast and prostate cancers remained relatively steady or declined since 2011. Our study showed an emerging trend of rapid increase of myeloid leukemia and CLL, but not all cancers, in the aging population of the United States.

The changes in leukemia diagnosis criteria could impact the incidences. Notably, WHO lowered the threshold of blast counts for AML diagnosis from 30% to 20% in its 3rd edition of classification in 2001^49^. That change could lead to an increase of AML incidence, which was consistent with a small spike of AML incidence in 2004 (Fig. 3b) given that the ACS data was a couple of years ahead of time. In contrast, AML incidence between years 2002–2004 in the SEER9 study was decreased after WHO criteria changes (Supplemental Fig. 1c), possibly due to the limited data in a smaller population. Nevertheless, such criteria changes were 10 years before the major aging-dependent leukemia trend changes described in this paper that is post-2011. Therefore, the vast majority of the impact of those WHO changes has already been factored in. The newest WHO classification, 5th edition in 2016^50^, has not yet affected ACS data collected in our analysis, and we don’t anticipate a major shift due to this newer revision.

One confounding factor that could contribute to the persistent increase in the AML rate is therapy-related AML (t-AML). t-AML is a clinical complication of cytotoxic chemotherapy or ionizing radiation therapy for malignant or non-malignant diseases, and it accounts for about 10–20% of all AML^17,51,52^. With the improvement in cancer patient survival, t-AML is on the rise^17,52^. However, ACS data were derived from multiple datasets that did not link individual cases and did not distinguish t-AML from *de novo* AML. These limitations prevent further separating the effect of t-AML from aging or more specific analysis of age effects. Using SEER datasets, several groups have reported that the risk for developing t-AML actually decreases with aging^53–55^. Given that opposite trend of t-AML with age and that t-AML represents only a small fraction of all AML, it is reasonable to believe that aging is still a major, and perhaps more important, contributor to the rapid increase of AML incidence.

Although the impact of aging “baby boomers” on cancer may not be fully realized until 2030 when all “baby boomers” turn 65^23^, it is a bit relief for now that the previously anticipated high rise of cancer incidences^24,25^ has not materialized. In this regard, ACS and SEER data were quite consistent: overall cancer incidence ceased to increase since 2011 in ACS data and decreased in SEER data. The precise mechanisms for this lack of rapid increase of all cancers with aging population remain to be investigated. We speculate that the decades’ efforts in cancer education, awareness, and prevention in the American public likely paid off. Many cancer prevention strategies including the cessation of smoking, reduced exposure to hazards such as radiation and toxic chemicals, ameliorated air and water pollution, and healthier diets and lifestyles may contribute to the reduction of overall cancer burden^56–58^. There is also epidemiological evidence linking smoking, certain diets and lifestyles to increased myeloid leukemia risk^59–61^. However, the incidences of myeloid leukemia continued to rise in both ACS and SEER data in general since 2011. Although the AML APC displayed a slight dip from 2015–2018 (Fig. 7) that warrants further monitoring, the dip is likely temporary as the AML incidents released by ACS in 2019 (after completion of our manuscript) already exceeded the previous peak in 2017 and the APC trend could resume climbing when more data become available. This raises an important question why myeloid leukemia rates are not reduced with the lower prevalence of common risk factors. A simple explanation may be that current preventive approaches may not be ideal or potent enough for reducing myeloid leukemia. Alternatively, aging may have a more prominent role in influencing myeloid leukemia through certain intrinsic mechanisms as detailed below.

Myeloid leukemia is derived from hematopoietic stem/progenitor cells. CML is caused by BCR-ABL1 transformation of hematopoietic stem cells (HSCs) whereas AML is derived from more committed myeloid progenitor cells^62^. All these cells reside in bone marrow and are physically shielded from extrinsic damages. Besides, the robust drug efflux activity of HSCs protects them from the damage of certain exogenous chemicals. However, these bone marrow-residing cells are still subjected to malignant transformation driven by cell-intrinsic mechanisms. One of such intrinsic mechanisms is HSC aging^63^. The quiescent HSCs along with committed bone marrow progenitor cells constantly replenish differentiated blood cells through hierarchical hematopoiesis, which is distinct from other organ systems such as heart and brain that rarely undergo tissue regeneration unless being damaged. Therefore, HSC aging may be distinct from the aging of other tissues and organ stem cells^64^. HSC aging is driven by many intrinsic factors and predisposes HSC themselves and committed myeloid progenitor cells for the accumulation of genetic and epigenetic alterations and formation of pre-leukemic clones that lead to eventual malignant transformation^63,64^. This may explain why the incidences of CML and AML are more likely influenced by aging. In contrast to myeloid leukemia, CLL is generally being considered a disease of differentiated mature B cells in the elderly. The age-dependent accumulation of genetic and epigenetic lesions leading to clonal B cell expansion underlies the molecular pathogenesis of CLL^65^. Intriguingly, a recent study indicated that the propensity to generate clonal B cells has already been acquired at the HSC stage in CLL patients^66^. Therefore, HSC aging can also contribute to CLL initiation and pathogenesis. We propose that the identification of new approaches to deter or inhibit HSC aging will ultimately help prevent and reduce myeloid leukemia and CLL. More concerted efforts will be needed to better understand the mechanisms of HSC aging and identify novel means for leukemia prevention in the future.

The rates of leukemia deaths, in general, reflect how effective the diseases are treated. CML is seen with the most dramatic decline of deaths reaching the lowest point in 2011. The substantial reduction of CML deaths is attributed to the effective treatment with the tyrosine kinase inhibitor imatinib mesylate that was approved by the FDA as the first line treatment for Philadelphia chromosome-positive CML in 2001. The remarkable effect of imatinib results in an ~80% 10-year survival rate of CML patients^67^. With imatinib and newer generations of tyrosine kinase inhibitors, CML patients are approaching normal life expectancies. It was projected that CML patient pool will further grow until a plateau is reached towards 2050^68^. However, imatinib and other tyrosine kinase inhibitors do not kill CML stem cells and the residual disease persists in most patients^69–72^. In addition, approximately 5 to 7% of CML patients eventually progress to blast crisis^10,73^. The current treatment options for CML blast crisis are limited and the outcome is dismal with a median survival of only 6–10 months^74,75^. With the expanding CML patient pool, it is expected that the absolute number of CML patients progressing to blast crisis will increase in the coming years even if the progression rate would remain steady. The resumed climbing of CML deaths after 2011 (Fig. 1d) may reflect such an increase in the CML patient pool. More effective treatments to eradicate CML stem cells and prevent disease progression are thus still needed to reduce CML deaths. Compared to CML, much less reduction of deaths is seen in other types of leukemia. The rate of CLL deaths has declined moderately in the past 20 years. Given that CLL incidence rate is increasing substantially, the moderate reduction of CLL deaths suggests that CLL treatments only partially place the disease under control^76^. The ALL death rate has remained constant and AML deaths continued to climb in the past 20 years. Therefore, much effort is needed to develop more effective therapeutics for these types of leukemia.

In conclusion, our study showed that the overall cancer incidence in recent years did not increase as rapidly as expected with the U.S. population aging in association with “baby boomers”, but the incidences of myeloid leukemia and CLL increased rapidly with a strong correlation with the population aging. Although the effect of “baby boomers” aging on cancer incidences remains to be fully realized in the next decade, the increase in myeloid leukemia and CLL appeared to significantly outpace that of all cancers. Before all “baby boomers” turn 65 in the year 2030^23^, the U.S. aging population will continue to grow, and leukemia incidences are expected to rise further. Therefore, this study will raise public awareness about these important trends of leukemia in the years to come, and provoke us to rethink cancer prevention strategies to reduce the incidences of leukemia and other cancers in aging Americans.

### Limitations

Due to the limited availability of information from ACS data, the authors were not able to further identify the exact causes of the correlations between population aging and cancer described in this paper, such as the state of health at 65, the mortality due to other causes, the effectiveness and availability of treatment, the rate of reporting, the accuracy of registries, the effect of races and age-specific rates. Although the results from ACS and SEER data showed certain similar trends, they were not quite the same and some were even opposite. The discrepancies could be due to the difference in population size and racial compositions that each dataset covers^77^, and thus direct comparison of them to match the results is difficult.

## Materials and Methods

### Data sources

For this study, we chose the cancer statistics data collected by ACS, because it covers the entire U.S. population. The incidences and deaths of leukemia and other cancers in the U.S.  from 1998 to 2018 were extracted from the annual Cancer Statistics data published by ACS^1,26–45^. According to ACS, the U.S. population-based cancer incidence data were collected by SEER program since 1973 and the Centers for Disease Control and Prevention’s (CDC’s) National Program of Cancer Registries (NPCR) since 1995^1^. These cancer incidence data were compiled and reported by the North American Association of Central Cancer Registries (NAACCR) since 1995^48^, which have approached 100% coverage of the US population in the recent time period and were the source for the projected new cancer cases by ACS^1^. The ACS projected cancer incidence data closely matched the final registered cases with about 10% variations^1,48^. All cancer cases were classified according to the International Classification of Diseases for Oncology except childhood and adolescent cancers, which were classified according to the International Classification of Childhood Cancer (ICCC)^78,79^. Mortality data from 1930 to 2015 were provided by the National Center for Health Statistics (NCHS)^1^. For this study, we chose ACS cancer statistics data from 1998 for our analysis because ACS entered a full-scale collaboration with NCI, CDC and NAACCR since 1998 for its annual cancer statistics reports^80^ and since then leukemia classification has conformed to WHO Classification of Tumors of Hematopoietic and Lymphoid Tissues^81^. For comparing ACS data with SEER data, we extracted SEER data from its 2018 and 2019 releases from the SEER online portal.

The total U.S. population and aging (65+) population were obtained from the published data by the U.S. Census Bureau. The total population-adjusted incidences and deaths of leukemia and other cancers were calculated by normalizing cancer incidences and deaths to the total population of the corresponding year. All graphs in this article were generated by GraphPad Prism or Microsoft Excel software, except for joinpoint trend analysis.

### Statistical analysis

Two-tailed Pearson r correlation analysis with 95% confidence interval was utilized to evaluate the relationship between each cancer type and the aging population over 65 years-old in the United States. Pearson r and p values were reported for each analysis performed with Prism software with p < 0.05 considered to be significant. Joinpoint regression analysis was carried out with log-linear model by using the SEER open-source software. Raw incidence was calculated by the number of cases per 100,000, and either the percent population over 65 or the year was used as the independent variable. The Monte Carlo Permutation method was used to test the statistical significance of trend changes with p < 0.05 considered to be significant.

## Supplementary information

Supplementary Info
