# Supplementary material for: An emerging trend of rapid increase of leukemia but not all cancers in the aging population in the United States
Source: Sci Rep. 2019 Aug 19;9:12070. doi: 10.1038/s41598-019-48445-1 (PMC6700310; doi:10.1038/s41598-019-48445-1)
Supplement: Supplementary file 1 — Supplementary Info [file 41598_2019_48445_MOESM1_ESM.pdf]

An emerging trend of rapid increase of leukemia but not all cancers in the aging population in the United States

Taisen Hao, PhD,[1] Min Li-Talley, PhD, [2] Alison Buck[3], and WenYong Chen, PhD[1,4].

[1]Department of Cancer Biology, Beckman Research Institute, City of Hope, Duarte, CA 91010.

[2]Department of Biostatistics, Beckman Research Institute, City of Hope, Duarte, CA 91010.

[3]Department of Biology, Biola University, La Mirada, CA 90638

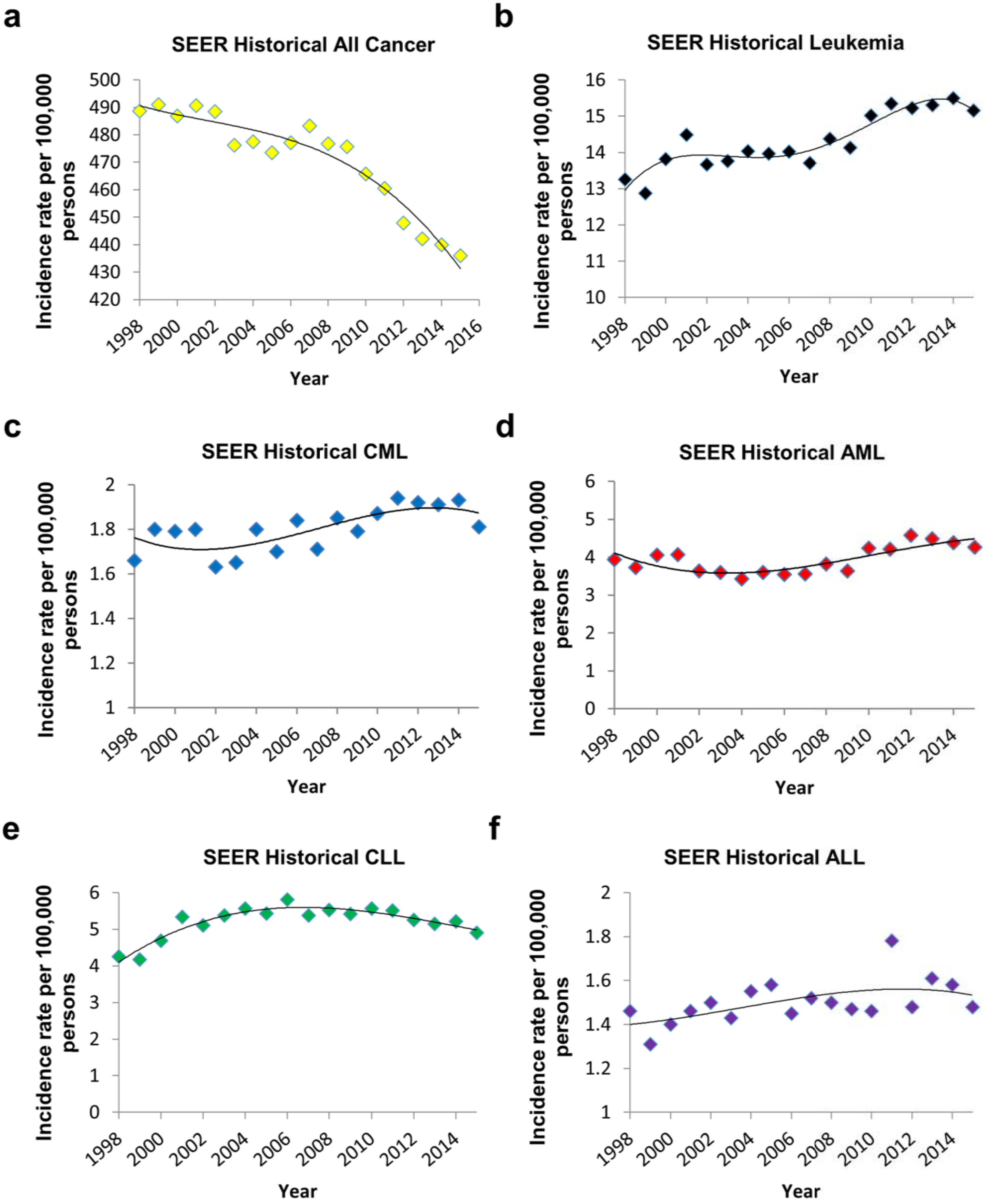

Supplemental Figure 1 Incidences of leukemia and all cancers by the SEER9 study.

Table S1 Influence of sex on the correlation of cancer incidences vs the percent population over 65

Cancer Type	1999-2011		2011-2018		Overall (1999-2018)	
	r-Value	p-Value	r-Value	p-Value	r-Value	p-Value
Female All Cancers	0.90	<0.0001	0.94	0.0006	0.94	<0.0001
Male All Cancers	0.84	0.0003	**-0.56**	**0.061**	0.70	0.0006
Female Breast Cancer	0.04	0.90	0.80	0.017	0.69	0.0007
Male Breast Cancer	0.77	0.0019	0.87	0.0048	0.86	<0.0001
Male Prostate Cancer	0.05	0.87	-0.94	0.0004	-0.58	0.007
Female Leukemia	0.73	0.0048	0.93	0.0009	0.90	<0.0001
Male Leukemia	0.80	0.001	0.97	<0.0001	0.93	<0.0001
Female CML	0.75	0.0032	0.93	0.0008	0.96	<0.0001
Male CML	0.23	0.45	0.91	0.002	0.91	<0.0001
Female AML	0.42	0.16	0.97	<0.0001	0.94	< 0.0001
Male AML	0.52	0.066	**0.69**	**0.059**	0.88	< 0.0001
Female CLL	0.75	0.0035	0.82	0.013	0.77	<0.0001
Male CLL	0.78	0.0018	0.79	0.020	0.81	<0.0001
Female ALL	0.84	0.0003	0.16	0.70	0.78	<0.0001
Male ALL	0.83	0.0004	-0.31	0.46	0.61	0.0047

Pearson correlation analyses were performed as in Table 1 for females and and males

Table S2 Comparison of joint point regression analysis of SEER leukemia data in 2015 and 2016

	JP Trend 1		JP Trend 2		JP Trend 3		AAPC (2006-16)	
**1975-2015 SEER leukemia incidence**	Years	APC	Years	APC	Years	APC	2006-15	2011-15
SEER 9 Delay-Adjusted Incidence, 1975-2015								
All Races	1975-99	0.2	1999-15	0.9			0.9	0.9
White	1975-99	0.3	1999-15	1.1			1.1	1.1
Black	1975-15	0.2					0.2	0.2
SEER13 Delay -Adjusted Incidence,1992-2015								
All Races	1992-15	0.6					0.6	0.6
White	1992-15	0.8					0.8	0.8
Black	1992-15	0.6					0.6	0.6
**1975-2016 SEER leukemia incidence**								
SEER 9 Delay-Adjusted Incidence, 1975-2016							2007-16	2012-16
All Races	1975-98	0.2	1998-16	0.8			0.8	0.8
White	1975-98	0.3	1998-16	1			1	1
Black	1975-16	0.3					0.3	0.3
SEER 21 Delay-Adjusted Incidence, 2000-2016								
All Races	2000-07	0.1	2007-11	2.7	2011-16	0.0	1.2	0
White	2000-08	0.5	2008-11	3.3	2011-16	0.0	1.2	0
Black	2000-07	-1.0	2007-16	2.2			2.2	2.2

2015 and 2016 SEER data were extracted from SEER Cancer Statistics Review released in 2018 and 2019, respectively.

The APC is the Annual Percent Change over the time interval.

Table S3 SEER21 cancer incidence trend from 2007 to 2016

	Cancer types	Total APC	Males APC	Females APC
All races	All sites	-1.3	-2.3	-0.3
	Breast	-0.1	0.3	0.1
	Prostate	-5.5	-5.7	–
	All leukemia	0.0	0.0	-0.1
	CML	1.3	1.1	1.4
	AML	1.6	1.8	1.2
	CLL	-0.9	-0.9	-1.0
	ALL	0.6	0.8	0.3
Whites	All sites	-1.3	-2.3	-0.4
	Breast	-0.2	0.0	-0.1
	Prostate	-6.0	-6.2	–
	All leukemia	-0.1	-0.2	-0.2
	CML	1.3	1.0	1.6
	AML	1.6	1.7	1.3
	CLL	-1.1	-1.1	-1.2
	ALL	0.4	0.8	-0.3
Blacks	All sites	-1.7	-3.0	-0.4
	Breast	0.2	1.5	0.4
	Prostate	-4.8	-5.2	–
	All leukemia	0.9	0.7	1.1
	CML	1.6	2.1	0.8
	AML	2.3	2.9	1.7
	CLL	0.6	0.2	0.7
	ALL	0	-1.4	2.4

SEER21 data were extracted from SEER Cancer Statistics Review 1975-2016 published in 2019.
The APC is the Annual Percent Change over the time interval.
